# Clinical manifestations and treatment of peripheral odontogenic keratocysts: two cases and a literature review

**DOI:** 10.1186/s13023-025-04087-3

**Published:** 2025-10-30

**Authors:** Yu Huang, Lirui Zhang, Xiaohong Yuan, Zhien Feng

**Affiliations:** 1 Department of Oral and Maxillofacial-Head and Neck Oncology, Beijing Stomatological Hospital, Capital Medical University, No.9 Fanjiacun Road, FengTai District, 100070 Beijing, China; 2 Department of Oral Pathology, Beijing Stomatological Hospital, Capital Medical University, No.9 Fanjiacun Road, FengTai District, Beijing, 100070 China

**Keywords:** Peripheral odontogenic keratocyst, Buccal mucosa, Microsurgical treatment, Review

## Abstract

**Background:**

Peripheral odontogenic keratocyst (POKC) is a rare extraosseous manifestation of odontogenic keratocyst (OKC). Because of their anatomical locations, OKCs and POKCs necessitate slightly different treatment approaches. However, the reported literature has not yet reached a definitive conclusion on this matter.

**Methods:**

This study included 2 patients who experienced postoperative recurrence of POKCs in the right buccal mucosa from 2018 to 2020. Additionally, we reviewed the reported cases and summarized the clinical features, treatment and prognosis of the POKCs.

**Results:**

The two patients with POKCs in this report experienced recurrence after the first surgery, and the second operation involved microscopic excision of the lesion with its adherent tissue. No recurrence was observed.

**Conclusions:**

Because POKCs are located in the soft tissue, they are difficult to remove and therefore prone to recur. In the treatment of POKCs, the surrounding adhesions can also be removed. Microscopic excision should be performed as well.

**Supplementary Information:**

The online version contains supplementary material available at 10.1186/s13023-025-04087-3.

## Highlights

- This is the first report on the primary and recurrence of POKCs in the buccal mucosa, with a long follow-up period.
- Complete excision is effective for POKCs, especially when the cyst wall is hard to separate from the surrounding tissue.
- Using a surgical microscope can improve separation accuracy and reduce damage to adjacent tissue.

**Supplementary Information:**

The online version contains supplementary material available at 10.1186/s13023-025-04087-3.

## Introduction

Odontogenic keratocysts (OKCs) are developmental odontogenic cysts with a high recurrence rate (12% ~ 50%) [1, 2]. The jaw, especially the mandible, is a common site of OKCs. Nevertheless, a very small proportion of OKCs involve soft tissue, leading to their reference as peripheral odontogenic keratocysts (POKCs). POKCs are prone to gingival and buccal mucosa with a male predominance. They are diagnosed on the basis of pathological features similar to those of OKCs.

Approximately 50 cases of POKCs have been reported in the literature. However, their treatment and prognosis have been poorly described. In our study, we included 2 POKC patients who were treated at Beijing Stomatological Hospital, Capital Medical University, between 2018 and 2020. Both patients have been followed up to the present. Moreover, we reviewed the reported cases to summarize the clinical characteristics, treatment and prognosis of POKC.

## Clinical report

### Case 1

A 38-year-old female patient with a right buccal mass and pain was admitted to the Beijing Stomatological Hospital, Capital Medical University. The mass first appeared 6 years ago, and a puncture examination was performed 3 years ago. The biopsy results suggested an epithelial cyst, but no other treatment was administered. At present, two round masses with maximum diameters of 2.0 cm and 1.5 cm were detected in the right buccal region. Computed tomography (CT) revealed a potentially benign solid mass in the anterior part of the right mandibular ascending branch. The patient underwent decompression with preservation of the parotid duct. A piece of irregular-shaped, ash grey soft tissue was collected for pathological examination, and soybean-like material was observed in the cyst cavity. The postoperative pathological results indicated a POKC. Following surgery, the patient was instructed to wear a cyst plug to maintain the patency of the cystic cavity. However, due to inappropriate placement and a lack of necessary adjustments, the patient ceased its use.

**Fig. 1:**
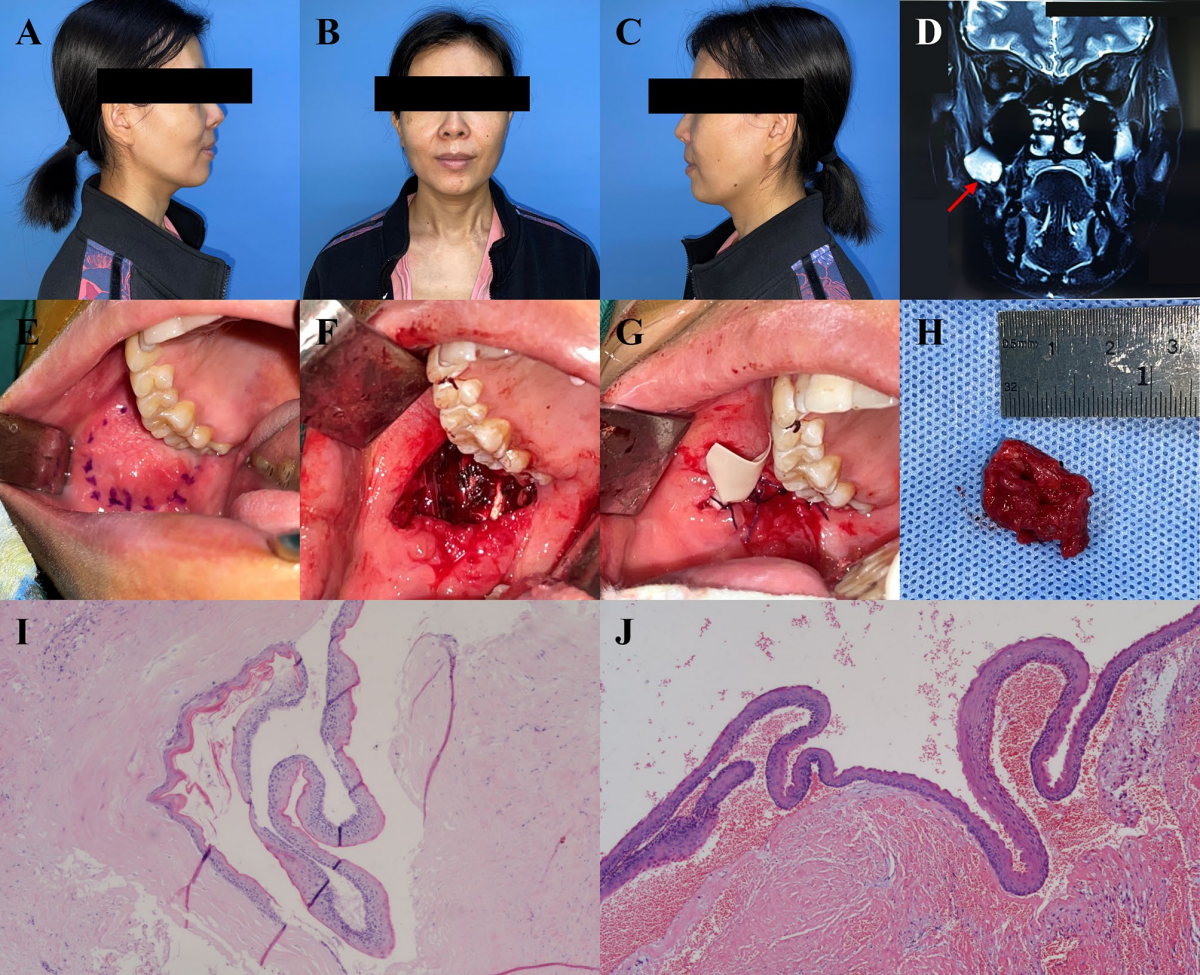
The clinical manifestations, surgical details, and postoperative pathology of case 1. (**A** ~ **C**) Front and side view of the case. (**D**) Enhanced MRI presented a cyst in the anterior upper part of the right mandibular ascending support. (**E**) Intraoral mark of the mass. (**F**) Completely resection of the mass under the surgical microscope. (**G**) A rubber strip was put for drainage. (**H**) Specimen of the excised mass. (**I** ~ **J**) Pathological section of H&E stain for the primary and recurrent surgery, respectively. Original magnification ×100

Approximately 3.5 years after surgery, the patient exhibited swelling and pain in the right buccal region, as well as moderate limitation of mouth opening. Local recurrence occurred four years after surgery. A solid cystic mass measuring 3.0 × 2.5 cm was observed in the posterior upper part of the right buccal mucosa. Enhanced magnetic resonance imaging (MRI) revealed a cyst in the anterior upper part of the right mandibular ascending branch, suggesting a recurrence. Complete resection was performed. During the operation, the cyst was found to be adhered to the surrounding submucosal and muscular tissues, especially the pterygomandibular ligament. Large amounts of white keratins were observed emerging from the cyst when the cyst wall was ruptured. Therefore, we completed excision under a surgical microscope. Postoperative pathology confirmed the POKC. No abnormalities were observed at the 37-month follow-up (Fig. 1).

### Case 2

A 38-year-old male patient was admitted to the Beijing Stomatological Hospital, Capital Medical University, for an asymptomatic right buccal mass that had been present for more than 6 years. The patient had previously undergone cyst incision and drainage in the outpatient department several times. Clinical examination revealed a round mass with a maximum diameter of 2.0 cm in the right buccal region. Enhanced CT (eCT) revealed a cystic lesion measuring 2.3 × 2.3 cm in the right temporal and external pterygoid muscles. Complete resection with preservation of the parotid duct was performed. A piece of irregular-shaped, ash grey-colored capsule wall tissue and irregular-shaped grayish yellow soft tissue were collected for pathological examination, and white keratin was found in the cyst cavity. Postoperative pathology confirmed the POKC.

**Fig. 2:**
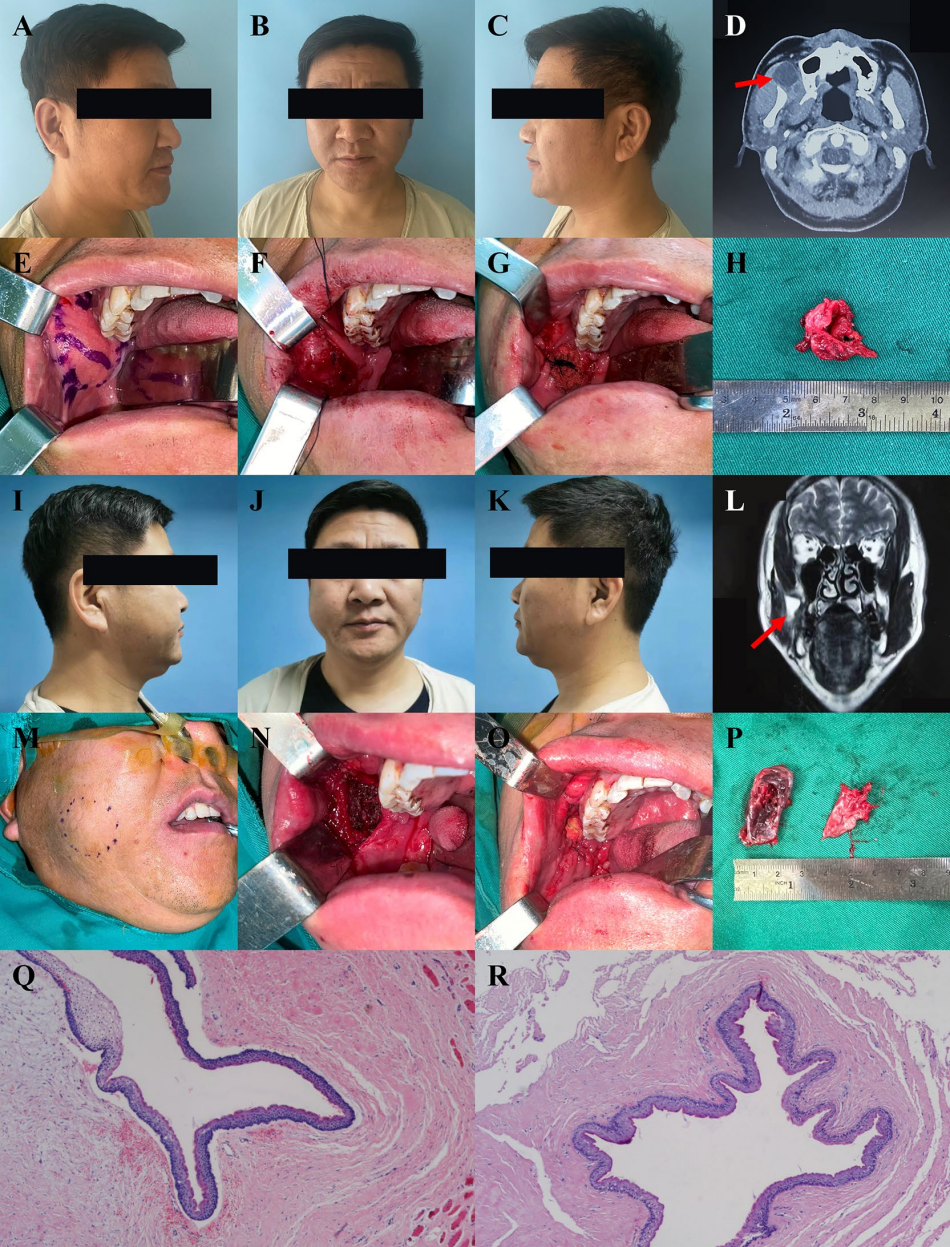
The clinical manifestations, surgical details, and postoperative pathology of case 2. (**A** ~ **C**) Front and side view before the primary surgery. (**D**) Maxillofacial enhanced CT showed a circular low-density nodule on the right side, between the temporal muscle and external pterygoid muscle. (**E**) Intraoral mark of the mass. (**F** ~ **H**) Procedure and postoperative specimen of the primary surgery. (**I** ~ **K**) Front and side view before the recurrent surgery. (**L**) Enhanced MRI showed a cystic space-occupying lesion anterolateral to the anterior ascending branch of the right buccal masseter. (**M**) Extraoral mark of the mass. (**N** ~ **P**) The cyst and part of the adhesive muscle were completely excised under the surgical microscope. (**Q** ~ **R**) Pathological section of H&E stain for the primary and recurrent surgery, respectively. Original magnification ×100

The mass recurred more than 3 years after the initial operation. The clinical findings were similar to those noted previously. MRI revealed a cyst attached to the masseter muscle anterolateral to the anterior ascending branch of the anterior masseter muscle of the right buccal muscle. During the operation, the cyst was found adhered to the surrounding submucosal and muscular tissues, especially the parotid duct, masseter muscle, and anterior ascending branch of the temporal muscle. The cystic wall was perforated during excision, emptying white keratin content. We completed the excision with a surgical microscope. Postoperative pathological results also confirmed POKC. No signs of recurrence were observed at the 16-month follow-up (Fig. 2).

## Results

The two POKC patients reported here both presented with lesions on the right buccal mucosa and experienced recurrence following their initial tumor resection. One patient underwent decompression and the other underwent cyst curettage first. During the second surgical procedure, the tumor along with the closely adhered surrounding tissue was meticulously excised under microscopic guidance. Two patients were followed up for 37 months and 16 months after the second surgery, and there was no recurrence in either case.

## Discussion

POKC is a particular type of OKC. Investigating the origin of POKC is critical, and its origin may potentially be deduced based on that of OKC. The most common theory suggests that OKCs arise from remnants of the dental lamina, which maintain their odontogenic potential and proliferate over time [3]. However, another hypothesis suggests that OKCs may originate from the basal cells of the oral mucosal epithelium, which is the origin of the dental lamina [4, 5]. These basal cells retain their ability for odontogenic differentiation and cause different lesions, such as peripheral ameloblastomas or OKCs, located within gingival tissues, especially in patients with Gorlin-Goltz syndrome [6–8]. Gorlin–Goltz syndrome or Nevoid basal cell carcinoma syndrome (NBCCS) is an autosomal dominant inherited condition comprising the principle triad of basal cell carcinomas, multiple jaw keratocytes, and skeletal anomaly [9]. Researchers have discovered that patents with Gorlin-Goltz syndrome have mutations in the PTCH1 gene, and the immunohistochemical results suggested that the OKC and POKC lesions were caused by a genetic alteration in the patients’ PTCH1-GLI gene [10]. The correlation between Gorlin–Goltz syndrome and POKC still needs to be further verified.

The clinical manifestations of POKC are nonspecific, often including soft tissue swelling. Patients may also exhibit restricted mouth opening if the mass involves adjacent structures such as the masticatory muscle [11]. The commonly used imaging examination methods include panoramic radiography, ultrasound, CT and MRI [12]. Strategies for treating and managing POKC have not been adequately documented thus far. In most studies on POKCs, the treatment modalities mainly include enucleation, curettage, and excision. In some cases, ostectomy, electrocautery, chlorhexidine rinses, and reconstruction with flaps were used [13]. Currently, the most common treatment methods for POKC are the same as those for OKC, and the main approach is curettage. This intraoral approach causes minimal trauma and allows complete lesion resection in one session [8, 14]. The recurrence rate of OKC treated by curettage alone is approximately 17% − 54.5%, mostly because of residual lesion [15, 16]. Marsupialization or decompression has been used as a more conservative form of treatment for a large OKC to minimize the cyst size and to limit the extent of surgery [8, 17]. Tucker et al. [18] reported a large mandibular OKC in a 15-year-old boy treated by decompression and secondary enucleation. Marker et al. [19] reported long-term results after decompression for 23 OKCs, and they concluded that these cysts could be treated successfully by decompression and subsequent enucleation.

A systematic literature review was performed by searching the Elsevier Journals, Web of Science, Wiley Online Library and PubMed databases for available case report articles on patients with POKC using the search terms “Peripheral odontogenic keratocyst”, “Keratocystic odontogenic tumor” and “Review” up to 2025. In addition, we performed the inspection of references of the identified articles, including case reports of peripheral tissue-originating cases that were clearly diagnosed as OKC and excluding articles not available in English, cases with unclear diagnoses or severe data deficiencies. We identified a total of 37 English articles, which collectively reported 51 cases, as detailed in Table 1. The review included 26 males and 22 females. The average age of the patients was 54 years (ranging from 14 to 83 years). Among them, 29 POKCs (56.9%) developed in the gingiva, and 14 (27.5%) involved the buccal mucosa. There were also 8 isolated cases where the onset location was in the temporal muscle (3), temporomandibular joint (1), masseter muscle (1), masticatory space (1), retromolar pad (1), and oropharynx (1). Among the reported cases of gingival POKC, 11 cases (37.9%) involved the mandibular gingiva, 14 cases (48.3%) involved the maxillary gingiva, 1 case involved both the maxillary and mandibular gingiva, and 3 cases had unknown involvement. Notably, 2 patients had POKCs in the gingiva as well as nevoid basal cell carcinoma syndrome (NBCCS). The chief complaint of 28 patients was swelling. Of these patients, 9 had pain, 4 had restricted mouth opening, 1 experienced discomfort while chewing, and 1 developed numbness. Two patients reported that they could feel the mass enlarging, and 1 of them felt that the mass affected appearance. One patient reported that a bite caused the mass.

**Table 1:** Clinical characteristic of reported cases of peripheral odontogenic keratocyst (POKC)

Case	Year	Author	Age	Gender	Site	Symptom	Size (cm)	Treatment	Recurrence	Recurrence-free time (months)	Retreatment
1	1975	Stoelinga et al. [20]	/	/	Maxillary gingiva	Accompanied by NBCCS disease	/	/	/	/	/
2	1979	Buchner and Hansen [21]	/	/	Gingiva	/	/	/	/	/	/
3			/	/	Gingiva	/	/	/	/	/	/
4	1988	Dayan et al. [22]	42	M	Left maxillary gingiva	Asymptomatic	1	Curettage	No	10	/
5	1992	Worrall SF et al. [23]	60	M	Left temporalis muscle	Swelling with pain and numbness	/	/	No (OKC occurred in the left mandible and coronoid process)	36	/
6	1994	Chehade et al. [24]	37	M	Right mandibulary gingiva	/	0.3	/	/	/	/
7			66	F	Left maxillary gingiva	/	/	/	/	/	/
8			70	M	Left mandibular gingiva	/	/	/	Yes	84	/
9			57	F	Right maxillary gingiva	/	0.7	/	/	/	/
10			42	M	Right mandibulary gingiva	/	/	/	/	/	/
11			35	F	Mandibular gingiva	/	1	/	/	/	/
12	1994	Fardal and Johannessen [25]	41	F	Mandibular and maxillary gingiva	/	/	/	/	/	/
13	2002	Ide et al. [26]	38	F	Left maxillary gingiva	Asymptomatic	0.3	Enucleation	No	60	/
14			46	F	Right maxillary gingiva	Asymptomatic	0.5	Resection	No	72	/
15	2005	Chi et al. [27]	81	F	Left mandibular gingiva	Asymptomatic	1	Enucleation, curettage	Yes	6	Excision
16			64	F	Left maxillary gingiva	Enlarging	1.5	Enucleation	No	21	/
17	2005	Preston and Narayana [28]	83	F	Left maxillary gingiva	Asymptomatic	0.7	Excision	No	6	/
18	2007	Mozaffari et al. [29]	82	F	Left mandibular gingiva	Swelling	0.7	Excision biopsy	/	/	/
19	2008	Ide et al. [30]	53	M	Left mandibular gingiva	/	0.6	/	No	84	/
20	2008	Faustino et al. [31]	57	F	Left mandibular gingiva	Swelling	0.5	Enucleation, curettage	Yes	12	Surgery
21	2009	Precheur and Krolls [32]	59	M	Right buccal space	Swelling with pain	3.1	Excision	/	/	/
22	2009	Jinbu et al. [33]	63	M	Left mandibular gingiva	Swelling with pain	1.5	Resection, curettage	No	12	/
23	2009	Eryilmaz T et al. [34]	67	F	Left temporomandibular joint	Swelling and restriction of mouth opening	4	Excision	No (3 surgeries were performed at the same location)	24	/
24	2010	Ide et al. [35]	60	M	Left buccal mucosa	Swelling	3	/	No	/	/
25			16	M	Right buccal mucosa	Bitten	0.5	/	/	/	/
26	2011	Vij et al. [36]	56	M	Left maxillary gingiva	Swelling	2.5	Excision	/	/	/
27	2012	Gröbe et al. [37]	52	M	Right buccal mucosa	Swelling	2	Excision	No	4	/
28	2013	Kaminagakura et al. [38]	37	M	Left buccal mucosa	Swelling accompanied by chewing discomfort	2	Excision	No	12	/
29	2013	Yamamoto et al. [39]	74	M	Right buccal mucosa	Swelling	5	Extirpated	No	48	/
30	2014	Abé et al. [40]	46	M	Left temporalis muscle	Swelling with pain	2.1	Excision	No	144	/
31	2014	Sakamoto et al. [10]	24	F	Mandibular gingiva	Accompanied by NBCCS disease	0.3	Excision	/	/	/
32	2014	Zhu et al. [41]	44	F	Left soft palate and pharynx	Asymptomatic	4	Extensive resection and submandibular gland flap repair	/	/	/
33			69	M	Right buccal mucosa	Swelling	2	Extensive resection and sternocleidomastoid and buccal pad flap repair	/	/	/
34	2015	Makarla et al. [42]	62	M	Right buccal space	Swelling and restriction of mouth opening	6	Excision	No	24	/
35	2017	Vazquez-Romero et al. [43]	32	M	Left maxillary gingiva	Enlarging and esthetic effection	0.4	Curettage	No	12	/
36	2019	Witteveen et al. [44]	63	M	Right buccal space	Swelling with pain	2.5	Excision	No	48	/
37			48	F	Left buccal space	Swelling	/	Excision	No	12	/
38	2019	Zhu Fengshuo [45]	58	M	Left temporalis	Swelling	4	Removed	No (Mandibular OKC has occurred)	/	/
39	2020	Rodrigues et al. [11]	43	F	Right maxillary gingiva	Swelling	1.5	Excision	No	48	/
40			63	F	Anterior mandibular gingiva	Swelling	1	Excision	/	/	/
41	2020	De Oliveira EM [46]	64	M	Right buccal space	Swelling	4.5	Excision	No	10	/
42	2020	Beena VT et al. [47]	61	M	Right buccal space	Swelling	4.5	Excision	No	6	/
43	2021	Adam Shathur et al. [48]	76	M	Right retromolar trigone	Swelling with pain	5	Excision	No	3	/
44	2021	Irene Lafuente-Ibáñez de Mendoza et al. [49]	61	F	Left maxillary gingiva	Asymptomatic	1	Excision	No	20	/
45			74	F	Left mandibular gingiva	/	1.5	Excision	No	48	/
46			14	M	Left maxillary gingiva	Asymptomatic	2	Excision	Yes	20	Excision
47	2021	Takuma Watanabe [50]	62	M	Right buccal space	Swelling with pain, restriction of mouth opening	1	Enucleation	Yes	1st:24, 2nd:6	1st: Enucleation 2nd: Excision
48	2022	Mustakim et al. [51]	57	F	Right masseter muscle	Swelling with pain, restriction of mouth opening	5	Excision, curettage	No	60	/
49	2023	Nabil Kochaji et al. [52]	17	M	Right buccal space	Swelling	2.7	Excision	No	6	/
50	2023	John K. Brooks et al. [53]	70	F	Right maxillary gingiva	Asymptomatic	0.4	Enucleation	Yes	1st:8 2nd: 6 3rd: suspected, 4	1st: curettage and peripheral ostectomy 2nd: curettage, electrocautery, chlorhexidine rinse and peripheral ostectomy
51	2023	María Hornillos-de Villota et al. [54]	58	F	Left masticatory space	Swelling	2.6	Cystectomy	No (Mandibular OKC has occurred)	3	/

Abbreviations/, not stated; F, female; M, male; NBCCS, nevoid basal cell carcinoma syndrome; OKC, odontogenic keratocyst.

By reviewing the literature, we found that enucleation, curettage, removal, resection, or decompression only removed the tumor, while excision typically involved removal part of the surrounding tissue. Excision in the case of adhesion is more invasive than others. In previous reports, 14 patients were treated with tumor removal alone [22, 26, 27, 31, 33, 34, 39, 43, 45, 53, 54], and 5 of them experienced recurrence after surgery [27, 31, 34, 45, 53]. 15 patients underwent excision and osteotomy directly with no recurrence after the operation [11, 28, 37, 38, 40, 43, 44, 46–49, 51, 52]. Furthermore, following the initial surgery, 2 patients experienced recurrence and no further recurrence was observed after subsequent excision procedures [27, 50]. Similarly, the two cases we presented here also recurred after the primary surgery, and there was no recurrence after subsequent excision.

Overall, in 34 patients whose follow-up records were available, 7 patients (20.6%) experienced recurrence [24, 27, 31, 34, 49, 50, 53]. In addition, 3 patients had previously developed mandibular OKCs [23, 45, 54]. The recurrence rate of POKCs in the gingiva was 31.25% (5 of 16) and that in the buccal mucosa was 9.1% (1 of 11). However, among all the patients followed up, only 11 were followed up for more than 3 years [11, 23, 24, 26, 30, 39, 40, 44, 49, 51], in which 2 patients with buccal POKCs and 6 patients with gingiva POKCs. Therefore, we believe that the recurrence rate of POKCs may be underestimated and that rate may be higher if patients can be followed up to more than 3 years. Our study is the second report on the recurrence of POKCs in the buccal mucosa, with a follow-up period of more than 50 months from the initial surgery. In Takuma Watanabe’s report, the cyst was not completely resected in the first two operations due to the large scope of the lesion and punctured cyst wall. At the third operation, the lesion was completely removed with the surrounding mucosal tissue and proximal bone. No recurrence was observed during the later follow-up. Based on observations from our two patients, we propose that two primary factors may compromise the therapeutic efficacy in POKCs. First, unlike intraosseous OKCs, POKCs are not easily decompressed due to insufficient soft tissue support, which compromises plug stabilization and often leads to unsatisfactory outcomes. Second, the frequent involvement of critical anatomical structures—such as the oropharynx, maxilla, and adjacent muscles—complicates complete excision. Simple enucleation in such cases increases the risk of leaving residual lesion, thereby elevating recurrence potential. Thus, complete excision is effective for POKC. Additionally, the use of a surgical microscope is also encouraged to improve separation accuracy and reduce damage to adjacent tissue.

Our report is by far the most comprehensive English review on POKC. Due to the rarity of POKC, all the reports were sporadic retrospective cases with limitations. Given the heterogeneity mentioned above, we believe that the current data is insufficient for a Meta-analysis. Although we adhered to structured search and reporting principles, the aim is to draw attention to the existing treatment strategies by presenting two rare cases, rather than providing evidence-based recommendations. We encourage the establishment of an international POKC registration system so that a comprehensive and systematic review can be conducted to assist doctors in exploring more effective treatment methods.

## Conclusion

We do not recommend simple tumor removal operations, such as enucleation or curettage. Excision, which involves the removal of the adhered tissue and the cyst, may be more effective to ensure the facial aesthetics and postoperative functional recovery for patients. Owing to their location, buccal POKCs in front of the pterygomandibular ligament and anterior temporalis muscle are prone to inflammation and adhesion. This region represents a high-risk site for recurrence, and surgery of cysts in such areas poses significant challenges. To avoid injury, sharp excision is recommended under the guidance of a surgical microscope. We also recommend extending the follow-up period for patients with POKCs to over 3 years and maintaining continuous monitoring of these cases to identify more effective treatment strategies that can significantly reduce the recurrence rate of POKCs.

## Supplementary Information

Below is the link to the electronic supplementary material.

Supplementary Material 1

## Data Availability

All data generated or analyzed during this study are included in this article.

## Abbreviations

POKC: Peripheral Odontogenic Keratocyst
OKC: Odontogenic Keratocyst
CT: Computed Tomography
MRI: Magnetic Resonance Imaging
eCT: Enhanced CT
NBCCS: Nevoid Basal Cell Carcinoma Syndrome
