# Supplementary material for: Clinical manifestations and treatment of peripheral odontogenic keratocysts: two cases and a literature review
Source: Orphanet J Rare Dis. 2025 Oct 30;20:550. doi: 10.1186/s13023-025-04087-3 (PMC12573988; doi:10.1186/s13023-025-04087-3)
Supplement: Supplementary file 1 — Supplementary Material 1 [file 13023_2025_4087_MOESM1_ESM.docx]

1. This is the first report on the primary and recurrence of POKCs in the buccal mucosa, with a long follow-up period.
2. Complete excision is effective for POKCs, especially when the cyst wall is hard to separate from the surrounding tissue.
3. Using a surgical microscope can improve separation accuracy and reduce damage to adjacent tissue.
